# Supplementary material for: Factors Influencing Dementia Care Competence among Care Staff: A Mixed-Methods Systematic Review Protocol
Source: Healthcare (Basel). 2024 Jun 6;12(11):1155. doi: 10.3390/healthcare12111155 (PMC11172285; doi:10.3390/healthcare12111155)
Supplement: Supplementary file 1 [file healthcare-12-01155-s001.zip › File S2. Documentation of search strategies.pdf]

Documentation of search strategies

Date: May 24, 2024

Topic/research question: Factors influencing dementia care competence among care staff: a mixed-methods systematic review protocol

---

Databases:

1. PubMed
2. Medline
3. Web of Science Core Collection
4. Embase
5. Scopus
6. Cochrane Library
7. CINAHL

Total number of hits:

- Before deduplication: 15,521
- After deduplication: 5,028

Comments:

1.PubMed

Date of Search: 2024-5-24<br>Number of hits: 3,212		
Search	Query	Records retrieved
#1	"dementia"[Title/Abstract] OR "Alzheimer"[Title/Abstract] OR "cognitive dysfunction"[Title/Abstract]	309,397
#2	"care staff*"[Title/Abstract] OR "nursing staff*"[Title/Abstract] OR "healthcare staff*"[Title/Abstract] OR "care worker*"[Title/Abstract] OR "care worker*"[Title/Abstract] OR "nurse"[Title/Abstract] OR "nurses"[Title/Abstract] OR "nursing"[Title/Abstract] OR "nursing personnel"[Title/Abstract] OR "licensed practical nurses"[Title/Abstract] OR "nursing assistant*"[Title/Abstract] OR "care assistant*"[Title/Abstract] OR "care aid*"[Title/Abstract] OR "medical specialist*"[Title/Abstract] OR "medical practitioner*"[Title/Abstract] OR "general practitioner*"[Title/Abstract] OR "doctor"[Title/Abstract] OR "doctors"[Title/Abstract] OR "physician"[Title/Abstract] OR "physicians"[Title/Abstract]	1,157,295
#3	"competency"[Title/Abstract] OR "competence"[Title/Abstract] OR "competences"[Title/Abstract] OR "knowledge"[Title/Abstract] OR "skill"[Title/Abstract] OR "skills"[Title/Abstract] OR "attitude"[Title/Abstract] OR "attitudes"[Title/Abstract] OR "challenge"[Title/Abstract] OR "judgment"[Title/Abstract]	1,855,786
#4	("dementia"[Title/Abstract] OR "Alzheimer"[Title/Abstract] OR "cognitive dysfunction"[Title/Abstract]) AND ("care staff*"[Title/Abstract] OR "nursing staff*"[Title/Abstract] OR "healthcare staff*"[Title/Abstract] OR "care worker*"[Title/Abstract] OR "care worker*"[Title/Abstract] OR ("nurse"[Title/Abstract] OR "nurses"[Title/Abstract] OR "nursing"[Title/Abstract] OR "nursing	3,212

	personnel"[Title/Abstract] OR "licensed practical nurses"[Title/Abstract] OR "nursing assistant*"[Title/Abstract] OR "care assistant*"[Title/Abstract] OR "care aid*"[Title/Abstract]) OR ("medical specialist*"[Title/Abstract] OR "medical practitioner*"[Title/Abstract] OR "general practitioner*"[Title/Abstract] OR "doctor"[Title/Abstract] OR "doctors"[Title/Abstract] OR "physician"[Title/Abstract] OR "physicians"[Title/Abstract])) AND ("competency"[Title/Abstract] OR "competence"[Title/Abstract] OR "competences"[Title/Abstract] OR "knowledge"[Title/Abstract] OR "skill"[Title/Abstract] OR "skills"[Title/Abstract] OR "attitude"[Title/Abstract] OR "attitudes"[Title/Abstract] OR "challenge"[Title/Abstract] OR "judgment"[Title/Abstract]) AND "English"[Language]	

2.Medline

Date of Search: 2024-5-24<br>Number of hits: 2,992		
Search	Query	Records retrieved
#1	dementia OR Alzheimer OR cognitive dysfunction (Abstract)	292,442
#2	"care staff*" OR "nursing staff*" OR "healthcare healthcare*" OR "care worker*" OR "care worker*" OR "nurse" OR "nurses" OR "nursing" OR "nursing personnel" OR "licensed practical nurses" OR "nursing assistant*" OR "care assistant*" OR "care aid*" OR "medical specialist*" OR "medical practitioner*" OR "general practitioner*" OR "doctor" OR "doctors" OR "physician" OR "physicians" (Abstract)	877,113
#3	"competency" OR "competence" OR "competences" OR "knowledge" OR "skill" OR "skills" OR "attitude" OR "attitudes" OR "challenge" OR "judgment" (Abstract)	1,741,538
#4	dementia OR Alzheimer OR cognitive dysfunction (Abstract) AND "care staff*" OR "nursing staff*" OR "healthcare healthcare*" OR "care worker*" OR "care worker*" OR "nurse" OR "nurses" OR "nursing" OR "nursing personnel" OR "licensed practical nurses" OR "nursing assistant*" OR "care assistant*" OR "care aid*" OR "medical specialist*" OR "medical practitioner*" OR "general practitioner*" OR "doctor" OR "doctors" OR "physician" OR "physicians" (Abstract) AND "competency" OR "competence" OR "competences" OR "knowledge" OR "skill" OR "skills" OR "attitude" OR "attitudes" OR "challenge" OR "judgment" (Abstract) AND English (Language)	2,992

3.Web of Science Core Collection

Date of Search: 2024-5-24<br>Number of hits: 2,729		
Search	Query	Records retrieved
#1	dementia OR Alzheimer OR cognitive dysfunction (Abstract)	281,760
#2	"care staff*" OR "nursing staff*" OR "healthcare healthcare*" OR "care worker*" OR "care worker*" OR "nurse" OR "nurses" OR "nursing" OR "nursing personnel" OR "licensed practical nurses" OR "nursing assistant*" OR "care assistant*" OR "care aid*" OR "medical specialist*" OR "medical practitioner*" OR "general practitioner*" OR "doctor" OR "doctors" OR "physician" OR "physicians" (Abstract)	703,704
#3	"competency" OR "competence" OR "competences" OR "knowledge" OR "skill" OR "skills" OR "attitude" OR "attitudes" OR "challenge" OR "judgment" (Abstract)	3,297,107
#4	dementia OR Alzheimer OR cognitive dysfunction (Abstract) AND "care staff*" OR "nursing staff*" OR "healthcare healthcare*" OR "care worker*" OR "care worker*" OR "nurse" OR "nurses" OR "nursing" OR "nursing personnel" OR "licensed practical nurses" OR "nursing assistant*" OR "care assistant*" OR "care aid*" OR "medical specialist*" OR "medical practitioner*" OR "general practitioner*" OR "doctor" OR "doctors" OR "physician" OR "physicians" (Abstract) AND "competency" OR "competence" OR "competences" OR "knowledge" OR "skill" OR "skills" OR "attitude" OR "attitudes" OR "challenge" OR "judgment" (Abstract) AND English (Language)	2,729

4.Embase

Date of Search: 2024-5-24<br>Number of hits: 958	Field tags:<br>· ti=Title<br>· ab= Abstract<br>· kw=Key Words<br>· la=Language

Search	Query	Records retrieved
#1	dementia:ti,ab,kw OR Alzheimer:ti,ab,kw OR 'cognitive dysfunction':ti,ab,kw	428,569
#2	'care staff*':kw OR 'nursing staff*':kw OR 'healthcare staff*':kw OR 'care worker*':kw OR 'nurse':kw OR 'nurses':kw OR 'nursing':kw OR 'nursing personnel':kw OR 'licensed practical nurses':kw OR 'nursing assistant*':kw OR 'care assistant*':kw OR 'care aid*':kw OR 'medical specialist*':kw OR 'medical practitioner*':kw OR 'general practitioner*':kw OR 'doctor':kw OR 'doctors':kw OR 'physician':kw OR 'physicians':kw	125,160
#3	'competency':ti,ab,kw OR 'competence':ti,ab,kw OR 'competences':ti,ab,kw OR 'knowledge':ti,ab,kw OR 'skill':ti,ab,kw OR 'skills':ti,ab,kw OR 'attitude':ti,ab,kw OR 'attitudes':ti,ab,kw OR 'challenge':ti,ab,kw OR 'judgment':ti,ab,kw	2,327,919
#4	(dementia:ti,ab,kw OR Alzheimer:ti,ab,kw OR 'cognitive dysfunction':ti,ab,kw) AND ('care staff*':kw OR 'nursing staff*':kw OR 'healthcare staff*':kw OR 'care worker*':kw OR 'nurse':kw OR 'nurses':kw OR 'nursing':kw OR 'nursing personnel':kw OR 'licensed practical nurses':kw OR 'nursing assistant*':kw OR 'care assistant*':kw OR 'care aid*':kw OR 'medical specialist*':kw OR 'medical practitioner*':kw OR 'general practitioner*':kw OR 'doctor':kw OR 'doctors':kw OR 'physician':kw OR 'physicians':kw) AND	958

	('competency':ti,ab,kw OR 'competence':ti,ab,kw OR 'competences':ti,ab,kw OR 'knowledge':ti,ab,kw OR 'skill':ti,ab,kw OR 'skills':ti,ab,kw OR 'attitude':ti,ab,kw OR 'attitudes':ti,ab,kw OR 'challenge':ti,ab,kw OR 'judgment':ti,ab,kw) AND English:la	

5.Scopus

Date of Search: 2024-5-24<br>Number of hits: 4,302	Field tags:<br>· ABS= Abstracts	
**Search**	**Query**	**Records retrieved**
#1	ABS (dementia OR Alzheimer OR "cognitive dysfunction")	328,932
#2	ABS ("care staff*" OR "nursing staff*" OR "healthcare staff*" OR "care worker*" OR "care worker*" OR "nurse" OR "nurses" OR "nursing" OR "nursing personnel" OR "licensed practical nurses" OR "nursing assistant*" OR "care assistant*" OR "care aid*" OR "medical specialist*" OR "medical practitioner*" OR "general practitioner*" OR "doctor" OR "doctors" OR "physician" OR "physicians")	1,177,431
#3	ABS ("competency" OR "competence" OR "competences" OR "knowledge" OR "skill" OR "skills" OR "attitude" OR "attitudes" OR "challenge" OR "judgment")	6,264,060
#4	(ABS (dementia OR Alzheimer OR "cognitive dysfunction")) AND (ABS ("care staff*" OR "nursing staff*" OR "healthcare staff*" OR "care worker*" OR "care worker*" OR "nurse" OR "nurses" OR "nursing" OR "nursing personnel" OR "licensed practical nurses" OR "nursing assistant*" OR "care assistant*" OR "care aid*" OR "medical specialist*" OR "medical practitioner*" OR "general practitioner*" OR "doctor" OR "doctors" OR "physician" OR "physicians")) AND (ABS ("competency" OR "competence" OR "competences" OR "knowledge" OR "skill" OR "skills" OR "attitude" OR "attitudes" OR "challenge" OR "judgment")) AND (LANGUAGE (English))	4,302

6.Cochrane Library

Date of Search: 2024-5-24<br>Number of hits: 593		
Search	Query	Records retrieved
#1	dementia OR Alzheimer OR "cognitive dysfunction" in Title Abstract Keyword	30,290
#2	nurse OR nurses OR "nursing personnel" OR "nursing staff" OR "nursing assistant" OR "healthcare professional" OR "licensed practical nurses" in Title Abstract Keyword	38,084
#3	"competency" OR "competence" OR "competences" OR "knowledge" OR "skill" OR "skills" OR "attitude" OR "attitudes" OR "challenge" OR "judgment" in Title Abstract Keyword	166,452
#4	"competency" OR "competence" OR "competences" OR "knowledge" OR "skill" OR "skills" OR "attitude" OR "attitudes" OR "challenge" OR "judgment" in Title Abstract Keyword AND dementia OR Alzheimer OR "cognitive dysfunction" in Title Abstract Keyword AND nurse OR nurses OR "nursing personnel" OR "nursing staff" OR "nursing assistant" OR "healthcare professional" OR "licensed practical nurses" in Title Abstract Keyword AND English in Language	593

7.CINAHL

<table>
<tr><td colspan="2">Date of Search: 2024-5-24
Number of hits: 733</td><td colspan="2">Field tags:
· AB= Abstracts
· LA=Language</td></tr>
<tr><td>Search</td><td colspan="2">Query</td><td>Records retrieved</td></tr>
<tr><td>#1</td><td colspan="2">AB dementia OR AB Alzheimer OR AB "cognitive dysfunction"</td><td>45,776</td></tr>
<tr><td>#2</td><td colspan="2">AB (nurse OR nurses OR "nursing personnel" OR "nursing staff" OR "nursing assistant" OR "healthcare professional" OR "licensed practical nurses")</td><td>208,431</td></tr>
<tr><td>#3</td><td colspan="2">AB ("competency" OR "competence" OR "competences" OR "knowledge" OR "skill" OR "skills" OR "attitude" OR "attitudes" OR "challenge" OR "judgment")</td><td>328,156</td></tr>
<tr><td>#4</td><td colspan="2">AB (dementia OR Alzheimer OR "cognitive dysfunction") AND AB (nurse OR nurses OR "nursing personnel" OR "nursing staff" OR "nursing assistant" OR "healthcare professional" OR "licensed practical nurses") AND AB ("competency" OR "competence" OR "competences" OR "knowledge" OR "skill" OR "skills" OR "attitude" OR "attitudes" OR "challenge" OR "judgment") AND LA English</td><td>733</td></tr>
</table>
